# What they do in the shadows: A low-cost imaging system for recording leaf expansion and movements

**DOI:** 10.1093/plphys/kiae189

**Published:** 2024-04-01

**Authors:** Alicja B Kunkowska

**Affiliations:** Assistant Features Editor, Plant Physiology, American Society of Plant Biologists; PlantLab, Institute of Life Sciences, Sant’Anna School of Advanced Studies, 56010 Pisa, Italy

Plants use sunlight to produce energy through photosynthesis. Leaves adjust their position throughout the day to harvest the optimal amount of sunlight and avoid photodamage. Leaf movements are controlled by changes in growth rates of different parts of the leaf: lamina, petiole, or both. Additionally, the petiole can bend either upward (hyponasty) or downward (epinasty) by changing the elongation rate between the abaxial and adaxial leaf sides (Küpers et al. 2023).

Hyponasty is a part of the shade avoidance syndrome (SAS), observed when plants compete for sunlight in dense vegetation (Casal 2013). Green leaves absorb most of the red light (R) while transmitting and reflecting most far-red light (FR). Thus, based on the F:FR ratio, plants can detect their neighbors. In the open sunny field, plants sense a high R:FR ratio (Pantazopoulou et al. 2017). The high amount of R light triggers phytochrome B protein (phyB) relocalization to nucleus. In nucleus, phyB suppresses phytochrome interacting factors (PIF) 4, 5, and 7, which are required for the hyponastic response (Pantazopoulou et al. 2017). In dense canopies, low R:FR ratio (more FR light) keeps phyB out of the nucleus, allowing PIFs to promote leaf elongation. However, the exact relationship between FR light intensity and leaf movement is still being studied.

To better understand the interplay between light perception and molecular regulation of leaf movement, we need to closely monitor how different parts of the leaf move. Several imaging systems are already available for studying leaf movement. For instance, one such system captures leaf movement using RGB time-lapse imaging (Rehman et al. 2020). The RGB imaging system depends on white light that affects photoreceptor activity, thus making it tricky to perform night-time imaging. Another consideration is where the cameras are placed. To distinguish petiole and lamina movements, it is necessary to acquire pictures from the side instead of from above, which can be achieved with 3D laser scanner–based leaf angle imaging methods (Michaud et al. 2017). However, these methods can be expensive and require specialized software. In this issue of *Plant Physiology*, **Lisa Oskam and colleagues (**Oskam et al. 2024**)** present a new, low-cost imaging system with an open-source image analysis pipeline.

In the system presented by Oskam and colleagues (Oskam et al. 2024), each camera captures 2 plants from a side view. These cameras are controlled by Raspberry Pi minicomputers, which store the images before transferring them to a separate desktop computer for analysis. The analysis of the captured pictures is facilitated by the open-source scripts written by Oskam and colleagues (Oskam et al. 2024). First, the pictures are cropped to separate the images of 2 individual plants. Then, the user selects 3 key orientation points on the first image: the leaf tip, the junction between the petiole and the lamina, and the rosette center. The latter point is also marked on the last captured picture. These selected points are then applied by the script to all subsequent images. Based on the points coordinates, the script employs triangulation between the meristem, petiole-lamina junction, and leaf tip to calculate the length and angles for the petiole, lamina, leaf, and junction (Fig. 1).

**Figure 1.**
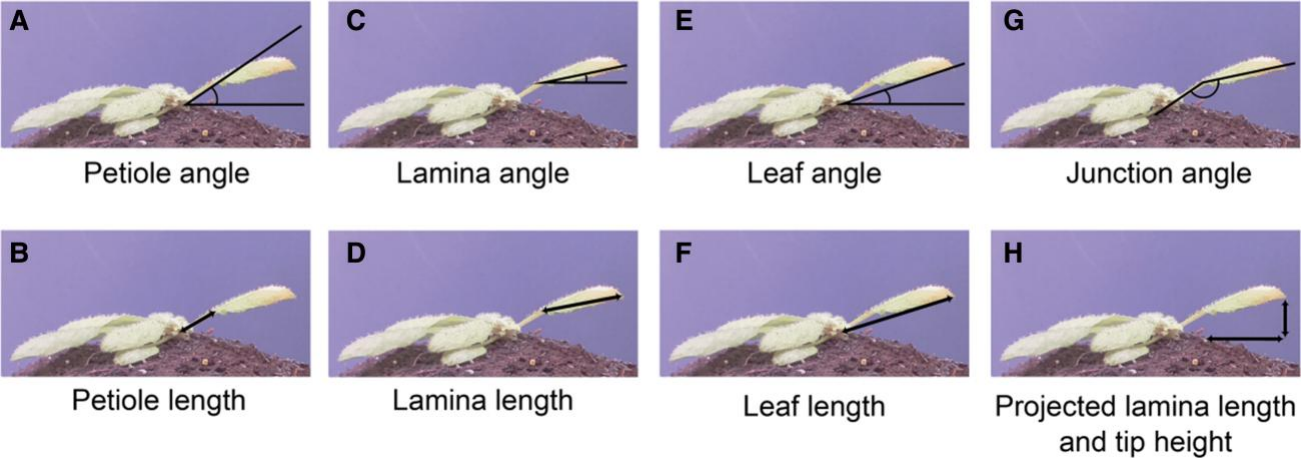
A visual representation of measurements conducted by the script, based on key orientation points: the rosette center, the petiole, and lamina tip. Adapted from (Oskam et al. 2024), Figure 2.

To evaluate the efficacy of their system, Oskam and colleagues (Oskam et al. 2024) performed a series of experiments to explore the relationship between FR light intensity and the magnitude of leaf movement. Their investigation focused particularly on elucidating potential differences between the movements of petiole and lamina. Initially, the authors subjected plants to either standard white light (WL) conditions or WL supplemented with consistent, whole-plant FR light (FRw). They observed that under WL conditions, both petioles and laminas move in a diurnal pattern, which is consistent with previous findings (Michaud et al. 2017; Woodley of Menie et al. 2019). This observation confirmed that the new system operates effectively without disrupting photoreceptor activity. However, the high temporal resolution of their novel system, which captures images every single minute, led to a new discovery. Oskam and colleagues (Oskam et al. 2024) found that FRw exposure specifically promoted petiole elongation while leaving lamina length unaffected.

In a subsequent experiment, the authors investigated the effects of increasing levels of FRw light intensity. Their findings not only validated the correlation between petiole elongation and FR intensity (Bongers et al. 2018) but also revealed a pattern: the correlation occurs only after several hours of the FR treatment. Initially, the petiole elongation responses were similar under different FR intensities, but after 8 h, petiole elongation was significantly higher under the higher FR intensities. This observation suggests that molecular regulatory mechanisms controlling early and late responses to FR light are distinct.

The authors proceeded to test the sensitivity of their system by performing experiments with *pif7*, *pif4pif5*, and *pif4pif5pif7* mutant lines, which were previously characterized for their reduced hyponasty responses (Michaud et al. 2017; Pantazopoulou et al. 2017). Notably, Oskam and colleagues (Oskam et al. 2024) revealed that *pif7* mutant initially responded to FRw similarly to wild type but lost the hyponasty at the end of the night. Furthermore, all 3 mutants exhibited a different regulation of hyponasty between the petiole and the lamina. This suggests that the junction between the lamina and the petiole may serve as an additional point of leaf positioning, with a yet unraveled molecular regulation.

The imaging system presented by Oskam and colleagues (Oskam et al. 2024) represents a great low-cost alternative, which can be easily implemented in many laboratories. With its improved spatial and temporal resolution, this system harbors the potential to shed light on molecular mechanisms controlling leaf elongation and movement. In the future, it would be interesting to explore the possible adaptations of this system for larger plant species such as crops like maze. The shift of energy resources from reproductive to vegetative growth, as observed during SAS, can potentially diminish crop yield. Better understanding and recording of all plant movements can help unravel the full molecular network involved in the plant movement kinetics. This, in turn, can contribute to increasing crop productivity.

## References

[kiae189-B1] Bongers FJ , Pierik R, Anten NPR, Evers JB. Subtle variation in shade avoidance responses may have profound consequences for plant competitiveness. Ann Bot. 2018:121(5):863–873. 10.1093/aob/mcx151 29280992 PMC5906909

[kiae189-B2] Casal JJ . Photoreceptor signaling networks in plant responses to shade. Annu Rev Plant Biol. 2013:64(1):403–427. 10.1146/annurev-arplant-050312-120221 23373700

[kiae189-B3] Küpers JJ , Snoek BL, Oskam L, Pantazopoulou CK, Matton SEA, Reinen E, Liao CY, Eggermont EDC, Weekamp H, Biddanda-Devaiah M, et al Local light signaling at the leaf tip drives remote differential petiole growth through auxin-gibberellin dynamics. Curr Biol. 2023:33(1):75–85.e5. 10.1016/j.cub.2022.11.045 36538931 PMC9839380

[kiae189-B4] Michaud O , Fiorucci AS, Xenarios I, Fankhauser C. Local auxin production underlies a spatially restricted neighbor-detection response in Arabidopsis. Proc Natl Acad Sci U S A. 2017:114(28):7444–7449. 10.1073/pnas.1702276114 28652343 PMC5514730

[kiae189-B5] Oskam L , Snoek BL, Pantazopoulou CK, van Veen H, Matton SEA, Dijkhuizen R, Pierik R. A low-cost open-source imaging platform reveals spatiotemporal insight into leaf elongation and movement. Plant Phys. 2024:195(3):1866–1879. 10.1093/plphys/kiae097 PMC11213255 38401532

[kiae189-B6] Pantazopoulou CK , Bongers FJ, Küpers JJ, Reinen E, Das D, Evers JB, Anten NPR, Pierik R. Neighbor detection at the leaf tip adaptively regulates upward leaf movement through spatial auxin dynamics. Proc Natl Acad Sci U S A. 2017:114(28):7450–7455. 10.1073/pnas.1702275114 28652357 PMC5514729

[kiae189-B7] Rehman TU , Zhang L, Wang L, Ma D, Maki H, Sánchez-Gallego JA, Mickelbart MV, Jin J. Automated leaf movement tracking in time-lapse imaging for plant phenotyping. Comput Electron Agric. 2020:175:105623. 10.1016/J.COMPAG.2020.105623

[kiae189-B8] Woodley of Menie MA , Pawlik P, Webb MT, Bruce KD, Devlin PF. Circadian leaf movements facilitate overtopping of neighbors. Prog Biophys Mol. 2019:146:104–111. 10.1016/j.pbiomolbio.2018.12.012 30597150

